# Clinical features of non-syndromic late developing supernumerary teeth: a sign of the third dentition?

**DOI:** 10.1186/s12903-024-04155-3

**Published:** 2024-03-28

**Authors:** Xiaoqing Li, Xu Gong, Min Yu, Xuemei Gao

**Affiliations:** Department of Orthodontics, Peking University School and Hospital of Stomatology, No. 22 Zhongguancun South Avenue, Haidian District, Beijing, 100081 P.R. China

**Keywords:** Supernumerary teeth, Epidemiology, Permanent dentition, Cone-beam computed tomography (CBCT), Development, Retrospective studies

## Abstract

**Objectives:**

This study aimed to summarize the clinical features of non-syndromic late developing supernumerary teeth (LDST) and comparisons with common supernumerary teeth (ST) and explore the association between LDST and the third dentition.

**Materials and methods:**

This study retrospected cone-beam computed tomography (CBCT) and medical history of 41,903 consecutive patients from January to December 2021. Comparisons between ST and LDST were evaluated by Chi-square test or Fisher exact test. Correlation between chronological age and dental stage age was evaluated by Spearman’s rank correlation coefficient. Binary logistic regression analysis was used to explore the features of LDST originating from the third dentition.

**Results:**

Sixty patients with 126 non-syndromic LDST and 1602 patients with 1988 non-syndromic ST were identified. The prevalence of ST and LDST was 3.82% and 0.14%, respectively, with a male-female ratio of 1.78:1 and 1.31:1. LDST patients mainly had LDST in multiple (58.33%) and bilaterally (41.67%), with an average of 2.1/patient. Most LDST were normal-shaped (84.13%), vertically oriented (71.43%), located in the mandible (80.16%), and distributed in the premolar region (82.54%). The study also indicated that the development of LDST was correlated with permanent teeth, with LDST developing 6.48 to 10.45 years later. In this study, 72.22% of LDST met the clinical criteria for the third dentition.

**Conclusions:**

LDST manifested different clinical features from common ST. LDST might be closely related to the third dentition.

**Clinical relevance:**

This work would help to comprehend LDST from a clinical perspective, and may be complementary to the criteria of the third dentition.

## Introduction

Supernumerary teeth (ST) refer to teeth or tooth-like structures in addition to the normal number of teeth [1]. The overall prevalence of non-syndromic ST is 0.2–0.8% and 0.5–5.3% in primary and permanent dentition, respectively, varying across ethnic groups [2]. Non-syndromic ST tend to be single, conical, inverted, impacted, and located in the maxilla [3]. Classic hypotheses concerning the etiology of ST include [4]: (i) atavism, ST labial/buccal to the deciduous dentition [5]; (ii) dichotomy theory, two teeth generated by one tooth germ that develop simultaneously [6]; (iii) environmental factors, and (iv) hyperactivity of dental lamina, ST that occur in addition to the permanent teeth, which are considered to stem from the third dentition/post-permanent dentition [7]. The anlage of third dentition develops lingually to all permanent tooth germs and regresses apoptotic before the eruption of permanent teeth [4, 7]. Therefore, teeth derived from the third dentition are located on the lingual/palatal side of permanent teeth with a shape similar to the preceding teeth [5, 6].

Non-syndromic late developing supernumerary teeth (LDST) refer to ST that develop obviously later than relevant teeth without features of relevant syndromes. The findings of LDST in previous studies were inconsistent; LDST could be single or multiple [8–10], unilateral or bilateral [11, 12], located in the maxilla or mandible or both jaws [13–15], detected in various regions including canine, premolar, and molar region [16, 17].

Most LDST were asymptomatic, usually detected during routine radiographic examinations. Complications including delayed eruption, malposition, and root resorption of adjacent teeth have been reported [18–20]. The etiology of LDST was unclear, and it was assumed to possibly originate from the third dentition [6]. Due to the insufficient cases reported thus far, it remains unknown regarding the criteria, prevalence, and clinical features of LDST in the population.

This retrospective study aimed to summarize the prevalence, radiographic features, and developmental stage of non-syndromic LDST. Furthermore, we compared LDST with common ST, and explored the association between LDST and the third dentition.

## Materials and methods

### Subjects

A consecutive case series of 41,903 patients who underwent cone-beam computed tomography (CBCT) at Peking University Hospital of Stomatology for dental or maxillofacial diseases from January to December 2021 were screened consecutively. CBCT and medical history were collected and analyzed. To be more specific, patients with deciduous or mixed dentition were often subjected to periapical film or orthopantomography examinations due to severe early childhood caries, facial and chin trauma, jaw cysts, unerupted teeth, or other conditions, through which supernumerary teeth were found. CBCT was taken to enhance diagnosis and treatment planning of surgical interventions afterwards. Among patients with permanent dentition, the reasons for taking CBCT were more complex and diverse, commonly including pre-orthognathic surgery assessments, cheek masses, jaw cysts, jaw osteomyelitis, and orthodontic evaluations for camouflage treatment with skeletal deformities. All CBCT scans were performed in accordance with standard ethical guidelines and clinical practices, either for examination purposes or to meet treatment needs.

**Fig. 1:**
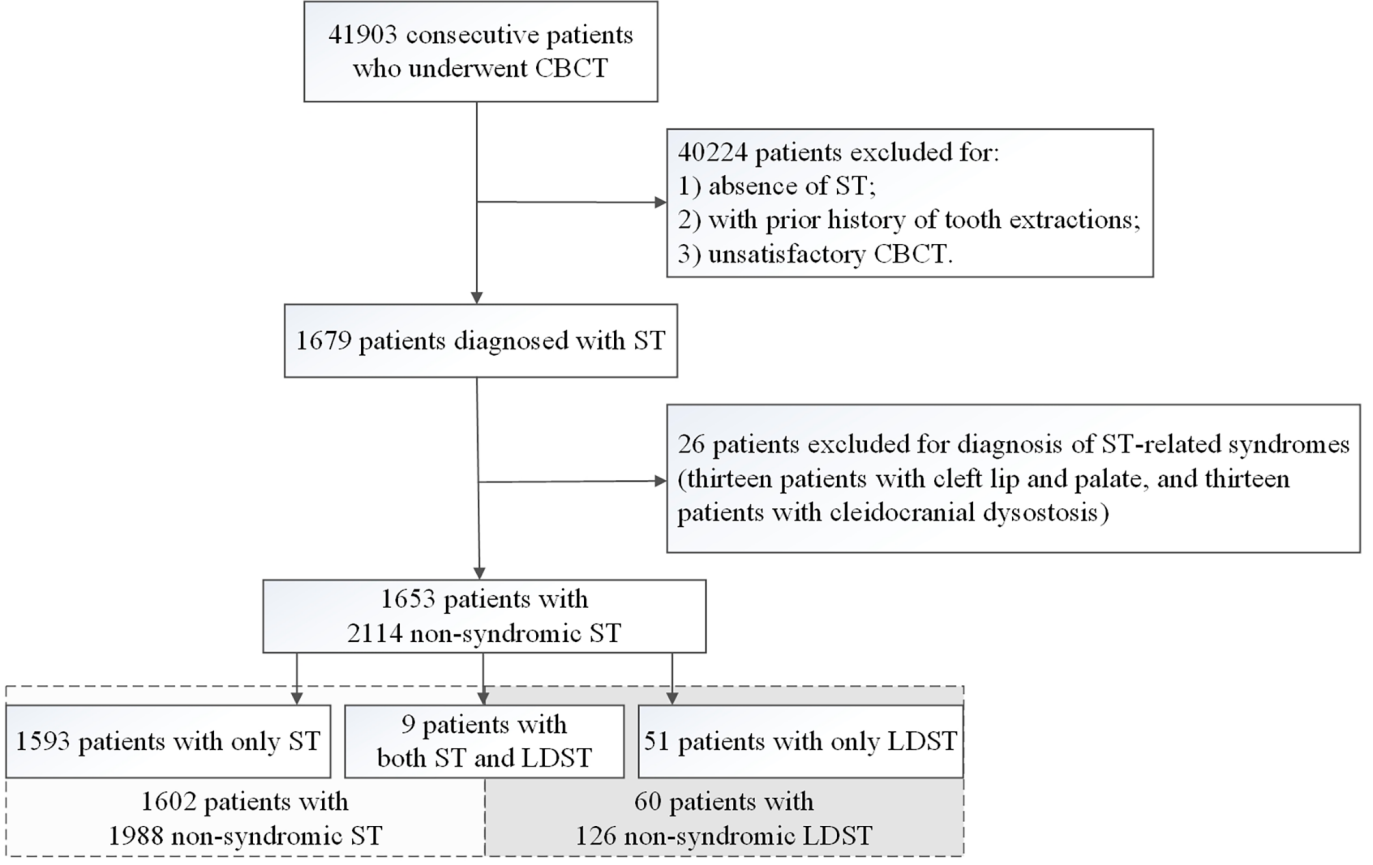
The flowchart of inclusion and exclusion of subjects. CBCT: cone-beam computed tomography; ST: supernumerary teeth; LDST: late developing supernumerary teeth;

The inclusion criteria were as follows: (i) clear CBCT images without artifacts around ST, and detailed medical records; (ii) presence with one or more ST; (iii) informed consent. The exclusion criteria included: (i) prior history of tooth extractions; (ii) diagnosed with systematic diseases; (iii) with ST-related syndromes such as Gardner’s syndrome, cleidocranial dysostosis, and cleft lip and palate. The present study finally enrolled 1953 patients with a total of 2114 non-syndromic ST (Fig. 1). This study conforms to STROBE guidelines.

### CBCT imaging and analysis

**Fig. 2:**
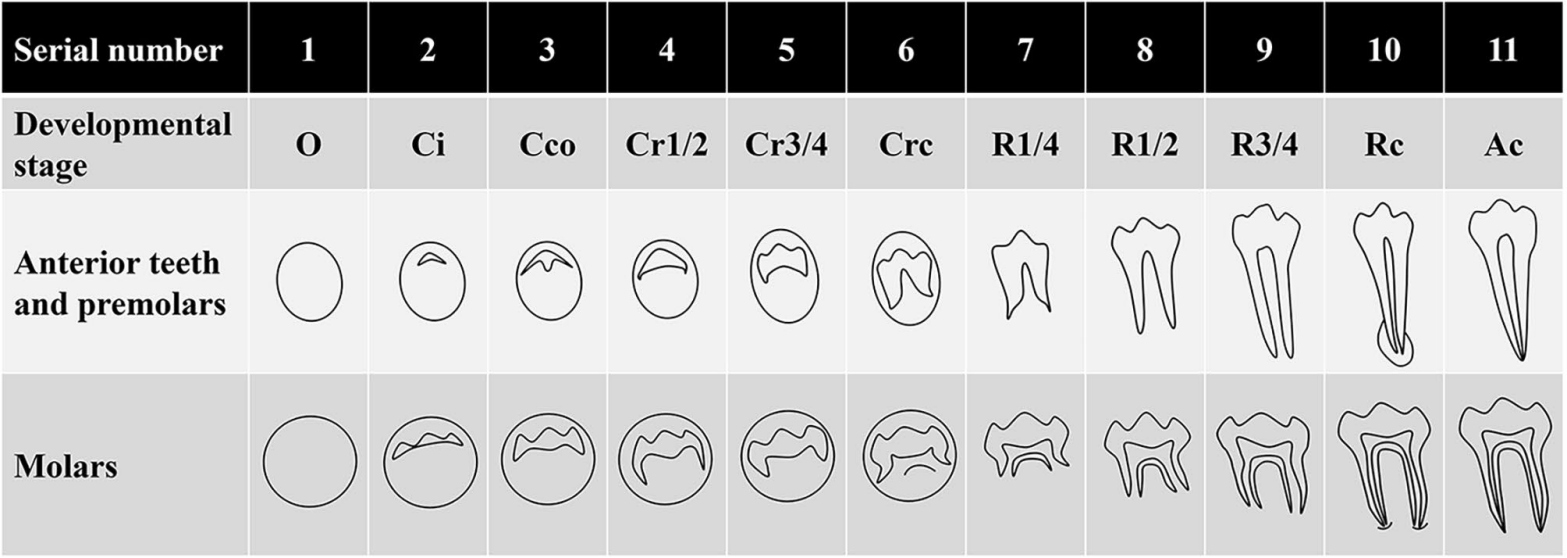
The developmental stages of LDST. O: crypt with no calcification; Ci: calcification initiation; Cco: coalescence of cusps; Cr1/2: crown 1/2 complete; Cr3/4: crown 3/4 complete; Crc: crown complete; R1/4: root 1/4 length; R1/2: root 1/2 length; R3/4: root 3/4 length; Rc: root complete; Ac: apex closed. The classification criteria were proposed by Kuremoto et al. [24]

All CBCT images were performed on patients with clinical demand and were taken under conventional technique and parameters using either DCT Pro (VATECH, Korea), NewTom VG (Quantitative Radiology, Italy), or i-CAT FLX (Imaging Science International, USA). The scanning protocol was as follows: DCT Pro with 90 kV, 7.0 mA, 24 s, FOV 20 cm × 19 cm; NewTom VG with 110 kV, 5.0 mA, 14.7 s, FOV 12 cm × 8 or 15 cm × 15 cm; i-CAT FLX with 120 kV, 5.0 mA, 18.5 s, FOV 16 cm × 13 cm. All the CBCTs were output in DICOM 3.0 format, and were assessed using Dolphin 18.0 (Dolphin Imaging &. Management Solution, USA). The images were reconstructed and analyzed in sagittal, coronal, and axial dimensions. The following analyses were performed for every ST and LDST:

i) The distribution of ST: central incisor, lateral incisor, canine, premolar, paramolar, distomolar;
ii) The position of ST crown: maxilla, mandible; unilaterality (midline, left side, right side), bilaterality; labial(buccal), within arch, lingual(palatal) [21];
iii) The shape of ST: conical, tuberculate, supplemental (normal), odontoma, undefined [22];
iv) The orientation of ST crown: normal, inclined, inverted, transverse, horizontal, undefined [21];
v) The state of eruption: erupted, impacted;
vi) The presence of complications of ST: none, cystic lesions, root resorption of adjacent teeth, impaction of adjacent teeth, malposition of adjacent teeth, rotation of adjacent teeth [23];
vii) The developmental stage of ST [24]: the eleven stages are shown in Fig. 2.

### Criteria for ST, LDST, and teeth from the third dentition

(i) ST: tooth or tooth-like structures beyond the 20 primary teeth or 32 permanent teeth [1, 3].
(ii) LDST: the diagnostic criteria for LDST are proposed in this study, either by the developmental stage or by chronological age. LDST are considered to be at least 3 stages behind the development of permanent teeth. Or the chronological age (CA) of LDST exceeds the dental stage age (DA) by at least 3 years [25]. Classification of developmental stages was proposed by Kuremoto et al., based on Haavikko’s method [26]. Chronological age referred to the age at the time of observation. Dental stage age was calculated based on normal developing age of permanent teeth, proposed by Kuremoto et al. as the average age of permanent teeth at specific developmental stage [24].
(iii) Teeth from the third dentition: (i) located on the lingual side of permanent teeth; (ii) with a similar shape to permanent teeth; and (iii) developed after permanent teeth are formed [4, 6, 27]. .

### Statistical analysis

Statistical analysis was performed with SPSS 23.0 (IBM Corp. Armonk, NY, USA). All the classifications were performed by one researcher and repeated twice after 14 days. If the results were inconsistent, an experienced orthodontist specialist was consulted. Self-repeatability for each variable was evaluated by Wilcoxon signed rank test, with a *p*-value ranging from 0.564 to 1.000. To access inter-observer variability and reliability coefficient, fifty ST patients were randomly selected and radiographic variables were evaluated by the two examiners independently, and ICC ranged from 0.847 to 0.986.

In this study, all the variables were not normally distributed based on normality test, thus, data were summarized as median (interquartile, IQR) and analyzed with nonparametric test. Spearman’s rank correlation coefficient was used to evaluate the correlation between chronological age (CA) and dental stage age (DA). Comparisons between ST and LDST were evaluated by the Chi-square test or Fisher exact test. Binary logistic regression analysis was used to explore the features of LDST originating from the third dentition. Statistical significance was considered with a *p*-value < 0.05.

## Results

### Epidemiological characteristics of ST patients and LDST patients

The present study enrolled a total of 1953 patients with 2114 non-syndromic ST in total (Fig. 1). ST were found in 1602 patients (median age 14.5 y, IQR 8.0, 30.0 y), and LDST were found in 60 patients (median age 17.0 y, IQR 13.0, 20.0 y). There were nine patients found with both ST and LDST. The prevalence of ST and LDST was 3.82% and 0.14%, respectively. Both ST and LDST were prominent in males, with a sex ratio of 1.78:1 and 1.31:1 (Table 1).

**Table 1:** Epidemiological data of 1602 patients with ST and 60 patients with LDST

Variable		ST patients *n* = 1062		LDST patients *n* = 60	
Age	3 ~ 5	62	3.87%	0	0%
	6 ~ 9	519	32.40%	0	0%
	10 ~ 17	274	17.10%	33	55.00%
	18 ~ 92	747	46.63%	27	45.00%
Dentition	Primary	64	4.00%	0	0%
	Mixed	588	36.70%	1	1.67%
	Permanent	950	59.30%	59	98.33%
Gender	Male	1026	64.04%	34	56.67%
	Female	576	35.96%	26	43.33%
Number	Single	1242	77.53%	25	41.67%
	Multiple	360	22.47%	35	58.33%
Arch	Maxilla	1471	91.82%	11	18.33%
	Mandible	126	7.87%	43	71.67%
	Both	5	0.31%	6	10.00%
Side	Midline	566	35.33%	0	0%
	Left	382	23.85%	22	36.67%
	Right	394	24.59%	13	21.67%
	Bilateral	260	16.23%	25	41.67%

### Comparison of ST and LDST

In this study, ST occurred in primary (4.00%), mixed (36.70%), and permanent (59.30%) dentitions, while LDST were found only in permanent dentition (Table 1). LDST differed from ST in number, morphology, orientation, and distribution (Table 2). The majority of ST patients (*n* = 1242, 77.53%) had a single ST, with 1.24 ST/patient. On the other hand, more than half of LDST patients (*n* = 35, 58.33%) had multiple LDST, with an average of 2.1 LDST. Moreover, LDST patients showed a greater tendency to form LDST on both sides (ST: 16.23%, LDST: 41.67%, *p*<0.001) and in both jaws simultaneously (ST: 0.31%, LDST: 10.00%, *p*<0.001).

In regard to morphology, conical was the most common shape of ST (*n* = 1281, 64.94%). Whereas, supplemental was dominant in LDST (*n* = 106, 84.13%). As for orientation, more LDST were in normal orientation (71.43%), in contrast to ST (16.50%), where most teeth were inverted (*n* = 665, 33.45%) or inclined (*n* = 597, 30.03%).

**Fig. 3:**
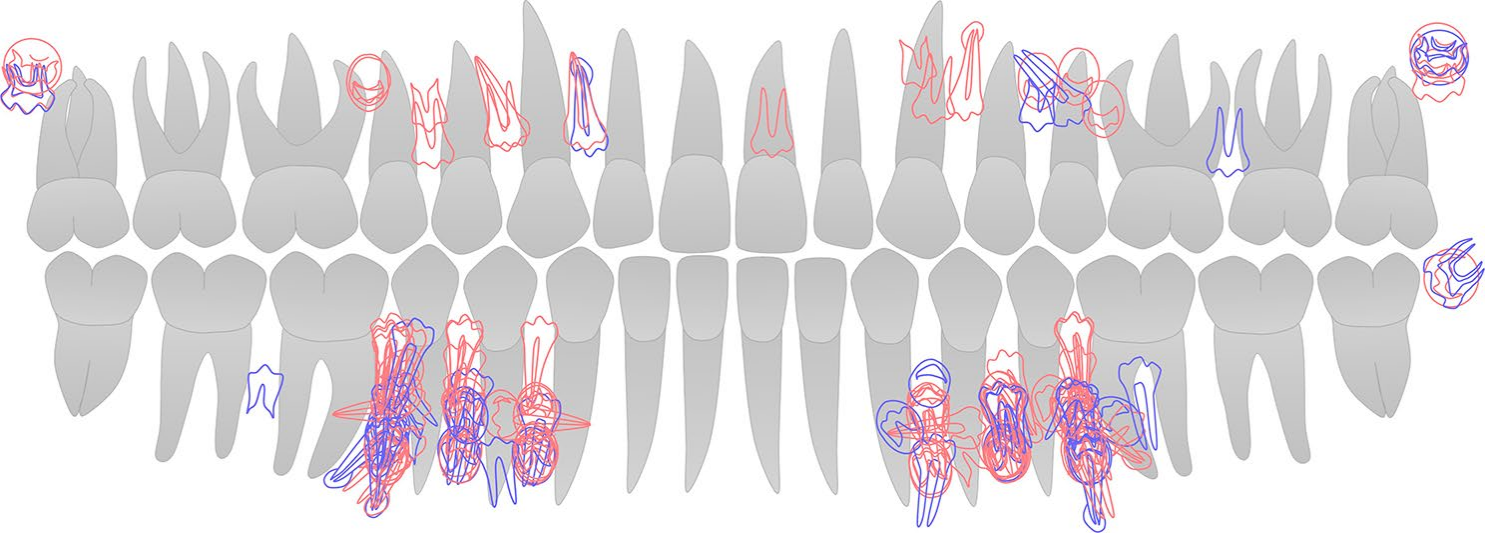
Overlapping map of the position, orientation, and developmental stage of 126 LDST. Red: male; blue: female. LDST were found in both jaws and occurred bilaterally. Predilection site is the premolar region in the mandible. The majority of LDST manifested a vertical orientation and normal shape similar to permanent teeth

With respect to distribution, most ST were found in the maxilla (*n* = 1837, 92.40%), while LDST were mainly in the mandible (*n* = 101, 80.16%). Mesiodens (*n* = 1472, 74.04%) were the most frequent type of ST, followed by supernumerary canines (*n* = 155, 7.80%) and lateral incisors (*n* = 118, 5.94%). LDST were found more in the premolar region (*n* = 104, 82.54%), followed by the distomolar region (*n* = 9, 7.14%). The detailed distribution of LDST is shown in Fig. 3. ST and LDST were impacted and asymptomatic in the majority, and the overall complication rate was 26.70% in ST and 11.11% in LDST.

**Table 2:** Comparison of radiographic characteristics between ST and LDST

Variable		ST (*n* = 1988)		LDST (*n* = 126)		*p*-value
Side ^a^	Unilateral	1435	72.18%	37	29.37%	< 0.001***
	Bilateral	553	27.82%	89	70.63%	
Arch ^a^	Maxilla	1837	92.40%	25	19.84%	< 0.001***
	Mandible	151	7.60%	101	80.16%	
Morphology ^b^	Conical	1281	64.44%	16	12.70%	< 0.001***
	Tuberculate	208	10.46%	3	2.38%	
	Supplemental	330	16.60%	106	84.13%	
	Odontoma	166	8.35%	0	0.00%	
	Undefined	3	0.15%	1	0.79%	
Localization ^a^	Labial/buccal	132	6.64%	8	6.35%	0.001**
	Median	525	26.41%	15	11.90%	
	Palatal/lingual	1331	66.95%	103	81.75%	
Orientation ^b^	Normal	328	16.50%	90	71.43%	< 0.001***
	Inclined	597	30.03%	27	21.43%	
	Inverted	665	33.45%	0	0.00%	
	Transverse	190	9.56%	5	3.97%	
	Horizontal	43	2.16%	2	1.59%	
	Undefined	165	8.30%	2	1.59%	
Developmental stage ^b^	Ac^1^	1632	82.09%	5	3.96%	< 0.001***
	Rc^2^	116	5.84%	18	14.29%	
	R3/4^3^	78	3.92%	13	10.32%	
	R1/2^4^	45	2.26%	20	15.87%	
	Before R1/2^5^	117	5.89%	70	55.56%	
Eruption status ^a^	Erupted	226	11.37%	14	11.11%	0.930
	Impacted	1762	88.63%	112	88.89%	
Complications ^b^	Asymptomatic	1569	78.92%	112	88.89%	0.007**
	Cystic lesion	44	2.21%	0	0.00%	
	Root resorption	27	1.36%	8	6.35%	
	Impaction	170	8.55%	5	3.97%	
	Malposition	57	2.87%	0	0.00%	
	Rotation	61	3.07%	0	0.00%	
	Fused tooth	5	0.25%	0	0.00%	
	Combination^6^	55	2.77%	1	0.79%	

** *p*<0.01; *** *p*<0.001. ^a^ Chi-square test; ^b^ Fisher’s exact test

1: Ac: apex closed; 2: Rc: root complete; 3: R3/4: root 3/4 length; 4: R1/2: root 1/2 length; 5: before root 1/2 length; 6: Combination: having more than one complication

### The developmental stage of LDST

**Fig. 4:**
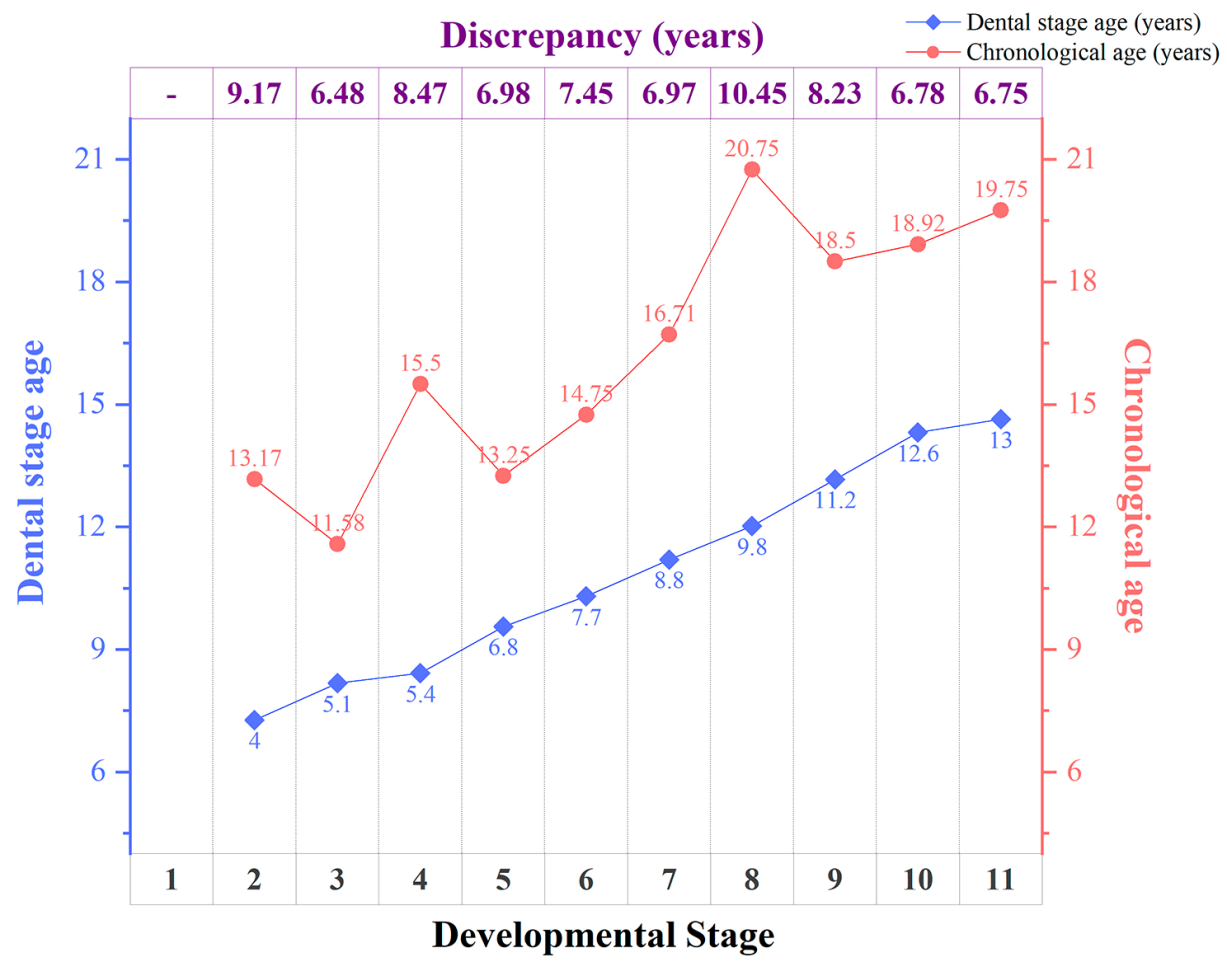
Chronological age (CA) and dental stage age (DA) of 126 LDST at each developmental stage. The numbers on the bottom refer to eleven developmental stages according to Kuremoto et al. The blue point represents the median dental stage age (DA), which refers to the normal developing age of permanent teeth at the corresponding developmental stage [24]. The red point represents the median chronological age (CA) of LDST at the specific developmental stage. The number at the top represents the median discrepancy between CA and DA for all LDST at each stage. Because no LDST at stage 1 was found, the corresponding position is denoted by “-”

No significant difference was found for age distribution at each stage between male and female, maxilla and mandible, left and right. The age difference between CA and DA for every single LDST ranged from 2.68 to 16.03 years (median age 7.34 y, IQR 5.85, 9.56 y). For all the LDST at a specific stage, the discrepancy between DA and CA ranged from 6.48 to 10.45 years. Figure 4 shows the relationship between age and developmental stage. The Spearman’s rank correlation coefficient was significant between CA and DA (*r* = 0.879, *p* = 0.001), suggesting a strong correlation between chronological age and dental stage age of LDST.

### Clinical features of LDST from the third dentition

Based on the aforementioned criteria, we identified that 91 LDST (72.22%) originated from the third dentition. Univariate comparisons of demographic characteristics and clinical features were performed between LDST that were attributed to the third dentition and those that were not. Variables that showed significant differences were then put into the binary logistic regression. The results showed that LDST numbered ≥ 3 (OR = 2.52; 95% CI, 1.13 to 5.59; *p* = 0.022), normal-oriented (OR = 2.99; 95% CI, 1.30 to 6.85; *p* = 0.008), and in the mandible (OR = 9.80; 95% CI, 3.67 to 26.18; *p* < 0.001) were more likely to originate from the third dentition. The *p*-value of the model was 0.018 and − 2log-likelihood ratio was 112.656. Though we observed LDST from third dentition more commonly seen in males, no statistical significance was demonstrated after multiple variate analysis.

## Discussion

ST, particularly LDST, are relatively rare conditions in clinical practice. According to previous literature, LDST could occur in males or females, single or multiple, unilateral or bilateral, and located in various regions of the maxilla or mandible. Limited by sample size, findings on LDST were inconsistent [28]. Since the third dentition was brought up [4, 6, 7], the research significance of LDST has become more obvious. This study attempted to characterize LDST based on a larger sample size and explore the association between LDST and the third dentition. To the best of our knowledge, this might be the most comprehensive study with the largest sample size on LDST.

In this study, the prevalence of LDST was 0.14% with a sex ratio of 1.31:1. As all the patients enrolled in this study were from a stomatological hospital, the sample population might not represent the overall population. Therefore, the prevalence of LDST may be overestimated. On the other hand, some LDST may have developed completely at the observation time and be classified as ST, leading to an underestimation of the prevalence according to the clinical criteria proposed in this study.

With respect to clinical features, most LDST were multiple, normal-shaped, normal-oriented, and bilaterally occurred in the mandible or both jaws. As they were mainly impacted, asymptomatic, and developed 6.48 to 10.45 years later than the corresponding permanent teeth, it’s recommended to take follow-up radiographs for early diagnosis and continuous monitoring [29]. Surgical removal should be considered when they were associated with pathological conditions or interfered with necessary treatment, such as causing delayed eruption of adjacent teeth or hindered space-closing during orthodontic treatment [19, 29–31]. In this study, LDST were found only in permanent dentition. One possible explanation is lack of longitudinal image data. It’s reasonable to suppose some LDST might have been undiagnosed and occasionally discovered later in permanent dentition with no symptom. Besides, some LDST in primary or mixed dentition, with normal teeth at early stages, could not meet the strict criteria, and therefore were grouped into ST.

Our study found that the clinical manifestations of LDST were much more similar to normally developed teeth than ST. The majority of LDST were present with characteristics consistent with teeth from the third dentition. And LDST with features including numbered ≥ 3, normal-oriented, and located in the mandible had a higher possibility to be originated from the third dentition. We found that the differences between the CA and DA of LDST at each developmental stage were relatively stable, with LDST developing 6.48 to 10.45 years later than the corresponding permanent teeth., which may be complementary to the criteria of the third dentition.

Although human dentition is diphyodont, multiple studies supported the ability of human dentition to form more than two generations of teeth [2, 4, 5]. It has been confirmed that ST could form as a result of the rescue of rudimentary teeth in animal models [32–34], and underlying molecular mechanisms have been explored in various animal models as well [35–37]. As for humans, some researchers have put up the idea that a third dentition might be locally stimulated to replace missing teeth [38]. And our results indicated a close association between LDST and the third dentition, which may be helpful for tooth regeneration in humans.

Our study has some limitations. Firstly, the clinical features found in this study were based on data from a single center. The epidemiological characteristics of LDST require further investigation. Secondly, this study was retrospective and observational, therefore, it may be biased by patients’ selection and missing information (such as incomplete family history). Thirdly, our study only included data over one year period. Some LDST might have developed completely by the time and be classified as ST. Therefore, it’s reasonable to suppose that the actual prevalence of LDST might be higher. Fourthly, the present study mainly focused on the characteristics of LDST clinically. Future studies are needed to elucidate the underlying mechanism.

## Conclusion

In conclusion, the present study investigated the clinical features of LDST based on a larger sample size. The developmental period of LDST was determined. And a close relationship between LDST and the third dentition was identified.

## Data Availability

The datasets used and/or analyzed during the current study are available from the corresponding author on reasonable request due to privacy reasons and large data size.
